# The role of chaperone‐mediated autophagy in neurotoxicity induced by alpha‐synuclein after methamphetamine exposure

**DOI:** 10.1002/brb3.1352

**Published:** 2019-07-09

**Authors:** Leping Sun, Yongling Lian, Jiuyang Ding, Yunle Meng, Chen Li, Ling Chen, Pingming Qiu

**Affiliations:** ^1^ School of Forensic Medicine Southern Medical University Guangzhou China; ^2^ Department of Anatomy Zunyi Medical College Zunyi China

**Keywords:** alpha‐synuclein, chaperone‐mediated autophagy, methamphetamine, neurotoxicity

## Abstract

**Introduction:**

Chaperone‐mediated autophagy (CMA) is an autophagy–lysosome pathway (ALP) that is different from the other two lysosomal pathways, namely, macroautophagy and microautophagy, and can selectively degrade cytosolic proteins in lysosomes without vesicle formation. CMA activity declines in neurodegenerative diseases such as Parkinson's disease, and similar neurotoxicity can occur after methamphetamine (METH) treatment. The relationship between CMA and METH‐induced neurotoxicity is not clear.

**Methods:**

We detected changes in the chaperone protein Hsc70 and the lysosomal surface receptor Lamp‐2a after METH treatment and then regulated these two proteins by small interfering RNA and DNA plasmid transfection to investigate how CMA influences METH‐induced neurotoxicity.

**Results:**

We found that CMA activity is decreased after METH exposure in neurons and downregulated Lamp‐2a can aggravate the neurotoxicity induced by α‐Syn after METH exposure and that Hsc70 overexpression can relieve the abnormal levels of alpha‐synuclein and its aggregate forms and the increase in cell apoptosis induced by METH.

**Conclusions:**

The results provide in vivo evidence for CMA plays a pivotal role in METH‐induced neurotoxicity, and upregulation of Hsc70 expression significantly protects neuronal cells against METH‐induced toxicity. This research may pave the way for potential therapeutic approaches targeting CMA for METH abuse and neurodegenerative disorders.

## 1 INTRODUCTION

According to the World Drug Report (2018), methamphetamine (METH) has become one of the most abused drugs in the world (https://dataunodc.un.org/drugs), and people who abuse METH long‐term may have brain damage similar to that in Parkinson's disease (PD) patients and an increased risk of developing PD (Callaghan, Cunningham, Sykes, & Kish, 2012; Curtin et al., 2015; Yu, Zhu, Shen, Bai, & Di, 2015). In‐depth studies have shown that the overexpression of alpha‐synuclein (α‐Syn) and other neurotoxic signs, such as oxidative stress, excitotoxicity, microglial activation, and dopaminergic neuron toxicity, are observed after METH treatment in vivo and in vitro (Mauceli et al., 2006; Leikin, 2012). Interestingly, α‐Syn is not only a major component of the pathological inclusions that are characteristic of several neurodegenerative disorders, such as Lewy bodies (LBs), which are observed in patients with PD (Recasens et al., 2014), but also plays a key role in neurotoxicity induced by METH (Chen, Huang, Wang, Qiu, & Liu, 2013). Under conditions of METH exposure, the increased generation of alpha‐synuclein has been found. Nevertheless, whether the α‐Syn degradation pathway is damaged remains unclear.

Chaperone‐mediated autophagy (CMA) is a selective catabolic pathway that allows the degradation of specific cytosolic proteins containing a pentapeptide targeting motif that is biochemically related to the KFERQ‐like sequence (Dice, 1990). This motif is selectively recognized by a constitutive chaperone, the chaperone heat shock cognate protein 70 kDa (Hsc70) (Chiang, Terlecky, Plant, & Dice, 1989). Hsc70, which resides in the lysosome (lys‐Hsc70), binds its substrate and targets the lysosomal receptor lysosome‐associated membrane protein type 2a (Lamp‐2a) to the lysosomal membrane (Agarraberes, Terlecky, & Dice, 1997; Cuervo & Dice, 1996); then, it translocates through the lysosomal membrane directly and is degraded in the lysosomal lumen (Bandyopadhyay, Kaushik, Varticovski, & Cuervo, 2008). As an eligible substrate, α‐Syn has been reported to be mainly degraded by CMA (Mak, McCormack, Manning‐Bog, Cuervo, & Di Monte, 2010; Vogiatzi, Xilouri, Vekrellis, & Stefanis, 2008), and CMA dysfunction has been identified as an important contributor to the pathogenesis of PD and α‐Syn aggregation (Cuervo, Stefanis, Fredenburg, Lansbury, & Sulzer, 2004; Malkus & Ischiropoulos, 2012). Herein, we focus on the relationship between CMA and α‐Syn‐induced neurotoxicity after METH treatment.

As mentioned above, there are two key elements in the CMA pathway, Hsc70 and Lamp‐2a. Lamp‐2a is considered to be the rate‐limiting step of the CMA pathway (Cuervo & Dice, 2000), the expression level of Lamp‐2a directly correlates with the CMA activity, and CMA levels can be modulated through the up‐ or downregulation of Lamp‐2a expression (Cuervo & Wong, 2014). Previous studies have shown that Lamp‐2a levels are decreased in α‐Syn inclusion‐forming regions of the substantia nigra and amygdala of PD patients (Alvarez‐Erviti et al., 2010), and the downregulation of Lamp‐2a increases endogenous α‐Syn levels in primary rat cortical neurons (Vogiatzi et al., 2008); on the contrary, Lamp‐2a overexpression reduces the level of monomeric α‐Syn, its phosphorylation and high‐molecular‐weight species (Xilouri et al., 2013). Hsc70 is not only the essential chaperone in the CMA translocation system that allows substrate protein entry into the lysosomal lumen (Cuervo & Wong, 2014) but also plays a critical role in neuronal death associated with neurodegenerative diseases due to its significant downregulation both in PD and AD (Alzheimer's disease) (Alvarez‐Erviti et al., 2010; Sala et al., 2014; Silva et al., 2014). Meanwhile, Hsc70 plays a key role in various cellular mechanisms, such as endocytosis, protein folding, and degradation (Stricher, Macri, Ruff, & Muller, 2013), and it binds both the soluble and fibrillar forms of α‐Syn and efficiently prevents its aggregation and propagation to limit the prion‐like cell‐to‐cell spreading of α‐Syn (Pemberton, Madiona, Pieri Kabani, Bousset, & Melki, 2011; Pemberton & Melki, 2012). Moreover, Hsc70 can effectively depolymerize α‐Syn fibrils into nontoxic monomers, thus relieving cytotoxicity (Gao et al., 2015).

To elucidate the role of CMA in METH‐induced neurotoxicity, we first assessed the changes in Lamp‐2a and Hsc70 expression levels after METH exposure using two cell lines, SH‐SY5Y cells and PC‐12 cells, and in primary neuronal cells from C57 fetal mice. In these three different types of cells, we found that Lamp‐2a expression showed a tendency to increase, while the levels of Hsc70 showed no obvious change, and α‐Syn and its aberrant forms were overexpressed, as previously reported (Vogiatzi et al., 2008). Based on this, we downregulated Lamp‐2a expression by small interfering RNA (siRNA), and the overexpression of α‐Syn and its abnormal aggregate forms were further increased as were the levels of apoptosis. We employed plasmid transfection to upregulate Hsc70, and this relieved α‐Syn‐induced neurotoxicity. Additionally, lysosomes containing both Lamp‐2a and lys‐Hsc70 can perform CMA (Cuervo, Dice, & Knecht, 1997), so we assessed CMA activity by analyzing the expression of Hsc70, Lamp‐2a, and Lamp‐1 by triple immunofluorescence labeling.

These findings demonstrate that CMA has a key role in the neurotoxicity induced by METH and that Hsc70 is an effective protective molecule for relieving cytotoxicity, suggesting that Hsc70 upregulation may represent a valuable potential therapeutic approach for METH abuse and neurodegenerative disorders.

## 2 MATERIALS AND METHODS

### 2.1 Cell culture

The human neuroblastoma SH‐SY5Y cell line and rat adrenal pheochromocytoma PC12 cell line were purchased from Shanghai Cell Bank of Chinese Academy of Sciences (Shanghai, China). These two cell lines from two different species (rats and humans) were selected for a parallel comparison of the findings of different studies and to determine whether the results we observed are species‐specific or relevant to both humans and other mammalian species (Huang et al., 2015; Qiao et al., 2014; Speen et al., 2015). The SH‐SY5Y cells and PC12 cells were cultured in DMEM and DMEM/F‐12, respectively, supplemented with 10% fetal bovine serum (Gibco) and maintained at 37°C in a humidified atmosphere containing 5% CO_2_. These cells were passaged every 2–3 days. The PC12 cells were initially exposed to 0, 1.0, 2.0, 2.5, 3.0, and 4.0 mM METH (>99% purity; National Institute for the Control of Pharmaceutical and Biological Products), and the SH‐SY5Y cells were exposed to 0, 0.5, 1.0, 1.5, 2.0, and 2.5 mM METH to evaluate the dose‐dependent expression of α‐Syn, Lamp‐2a, and Hsc70. METH concentrations of 3.0 mM for the PC12 cells and of 2.0 mM for the SH‐SY5Y cells were selected for subsequent experiments based on the LC25s of METH in these two cell types and the significant alterations in the levels of α‐Syn‐, CMA‐, and apoptosis‐related marker proteins at these concentrations (Chen et al., 2013, 2016; Huang et al., 2015; Xu et al., 2017). Next, we exposed the PC12 cells and SH‐SY5Y cells to 3.0 or 2.0 mM METH, respectively, for 0, 2, 4, 8, 16, and 24 hr to evaluate the time‐dependent changes in the expression of α‐Syn, Lamp‐2a, and Hsc70 after METH treatment.

### 2.2 Primary neuronal culture

Primary prefrontal cortex and striatal neuronal cells were cultured as described previously (Dong et al., 2012; Xu et al., 2017). Briefly, the prefrontal cortex and striatal neuronal tissues of C57BL/6J mice embryos were isolated from pregnant C57 mice (Laboratory Animal Center of Southern Medical University, Guangzhou, China) on days 16–18 of pregnancy by cesarean section and drenched in cold phosphate‐buffered saline (PBS) solution. Then, the prefrontal cortex and striatal tissues were minced into 1‐mm^3^ pieces and collected in a 15‐ml falcon tube. The tissue homogenates were digested at 37°C in a humidified atmosphere containing 5% CO_2_ by adding 5 ml 0.25% trypsin‐EDTA (Gibco) for 15 min, and the digestion was terminated by adding 15 ml DMEM/F12 supplemented with 10% FBS. Then, the cells were centrifuged for 5 min at 1,000 *g* and resuspended in 10 ml neurobasal medium containing 2% B27 (Gibco), 1% Glutamax‐100X, and 1 mM glutamate (Gibco). Then, the cells were plated in six‐well plates (1–2 × 10^6^ cells per well) or confocal dishes (2–4 × 10^5^ cells per well), which were precoated with 0.01% poly‐l‐lysine (Sigma). Half of the medium was replaced 3 days after plating and every 2 days thereafter, and the neuronal cultures were subjected to Western blot and immunofluorescence analyses one week after plating.

### 2.3 Western blotting

SH‐SY5Y cells, PC12 cells, and primary neuronal cultures were lysed in ice‐cold RIPA buffer with protease inhibitors and phosphatase inhibitors at 4°C for 30 min, and the protein concentrations were determined using a Bicinchoninic Acid (BCA) Protein Quantitative Analysis kit (Biocolors). An equal amount of protein from each sample was loaded on 8%–12% sodium dodecyl sulfate polyacrylamide gels (SDS‐PAGE) for electrophoresis and transferred onto PVDF membranes (Millipore). Then, the membranes were incubated at room temperature for 2 hr in blocking buffer (5% nonfat dry milk or 5% BSA in TBST buffer) followed by incubation with diluted primary antibodies overnight at 4°C with gentle shaking. Anti‐Lamp‐1 (lysosome marker; ab24170; 1:1,000 dilution), anti‐Lamp‐2a (ab18528; 1:1,000 dilution), and anti‐Hsc70 antibodies (13D3; ab2788; 1:1,000 dilution) were purchased from Abcam, Inc. Anti‐α‐synuclein (#2628; 1:1,000 dilution), anti‐phospho‐α‐synuclein (#23706; 1:1,000 dilution), anti‐cleaved caspase‐3 (#9661; 1:1,000 dilution), and anti‐PARP antibodies (#9532S; 1:1,000 dilution) were obtained from Cell Signaling Technology, Inc. An anti‐aggregated α‐Syn antibody, clone 5G4 (#MABN389; 1:1,000 dilution), was purchased from Millipore, Inc. After that, the membranes were washed three times with TBST and then incubated with HRP‐conjugated secondary antibodies (1:10,000) for 1 hr at room temperature. The membranes were washed as described above and then developed with chemiluminescence reagents (Thermo Scientific), and the band signal intensities were quantitated with a Gel‐Pro analyzer (Media Cybernetics, Inc.). The levels of beta‐actin (Cat. #bs‐0061R, Bioss) were utilized as a qualitative control.

### 2.4 Triple immunofluorescence labeling

To determine the colocalization level of Hsc70 with Lamp‐2a and Lamp‐1, we performed triple immunofluorescence labeling on the cells seeded on confocal dishes. The cells were fixed with 4% paraformaldehyde for 15 min, permeabilized with 0.1% Triton X‐100 (Amresco, diluted in PBS) for 3–5 min, blocked with 10% bovine serum albumin (Solarbio, diluted in PBS) for 1 hr at room temperature, and then incubated with diluted primary antibodies overnight at 4°C. The antibodies used were a rabbit anti‐Lamp‐2a antibody (1:200, diluted in PBS; Abcam), a mouse anti‐Hsc70 antibody (1:200, diluted in PBS; Abcam), and a rat anti‐Lamp‐1 antibody (1:200, diluted in PBS; Santa Cruz). After the samples were washed with PBST (1× PBS and 0.1% Tween‐20) three times, they were incubated with the following fluorescent secondary antibodies at room temperature for 1 hr: DyLight 405‐labeled goat anti‐rabbit IgG (1:100, diluted in PBS; Beyotime), FITC‐labeled goat anti‐mouse IgG (1:200, diluted in PBS; Beyotime), and Cy3‐labeled goat anti‐rat IgG (1:200, diluted in PBS; Beyotime). After washing as described above, anti‐fluorescence quenching mounting medium was added, and microphotographs were taken using a laser confocal fluorescence microscope (A1+/A1R+, Nikon, Tokyo, Japan). All digital images were processed using the same settings to improve the contrast. And we used Carl Zeiss ZEN 2.1 software to quantify the fluorescence intensity of each protein in the original image and optimize its fluorescence intensity at the same level to be suitable for observation.

### 2.5 TUNEL staining

Cell apoptosis was detected using an In Situ Cell Death Detection Kit, POD (Roche Applied Science) according to the manufacturer's instructions. The cells were fixed with 4% paraformaldehyde for 15 min, incubated with the TUNEL reaction mixture at 37°C for 1 hr in the dark, mounted with DAPI (40,60‐diamidino‐2‐phenylindole) for nuclear counterstaining, and imaged (10 × objective) using a fluorescence microscope (Nikon). The number of TUNEL‐ and DAPI‐positive cells in at least 3 fields was counted. The data are presented as the TUNEL index, which was calculated based on the total number of TUNEL‐positive cells.

### 2.6 Small interfering RNA/overexpression plasmid and transfection

A Lamp‐2a small interfering RNA and an Hsc70 overexpression plasmid for SH‐SY5Y cells were synthesized by Gene Pharma. The sequence of the Lamp‐2a siRNA was (homo: 5′‐GGCAGGAGUACUUAUUCUATT‐3′), and the sequence of the Hsc70 plasmid DNA was (homo: 5′‐ACGGGCCCTCTAGACTCGAGATGTCCAAGGGACCTGCAGTTG‐3′). Cells were seeded in a 6‐well plate and were grown to 70%–90% confluence at the time of transfection. A total of 2,500 ng of plasmid DNA, 5 µl of P3000 reagent, and 5 µl of Lipofectamine 3,000 reagent (Invitrogen) were mixed with Opti‐MEM medium (Gibco BRL) for plasmid transfection, and 20 nmol siRNA was diluted to transfect the cells following the protocol described for DNA excluding the addition of the P3000 reagent. The mixed solution was incubated for 15 min at room temperature, and the cells were incubated with this mixed solution for 6 hr. After that, the complex medium was replaced with complete medium for a 24‐hr incubation period prior to treatment with METH.

### 2.7 Statistical analyses

All data are expressed as the means ± standard deviations (*SD*) of at least three independent replicates. Statistical analysis was performed using one‐way ANOVA followed by least significant difference (LSD) *post hoc* analysis or independent samples *t* test (as appropriate) using the scientific statistics software SPSS version 19.0 (SPSS Inc.). A value of *p* < 0.05 was considered statistically significant.

## 3 RESULTS

### 3.1 METH increases the expression of α‐Syn and its abnormal aggregate forms in neurons

**Figure 1:**
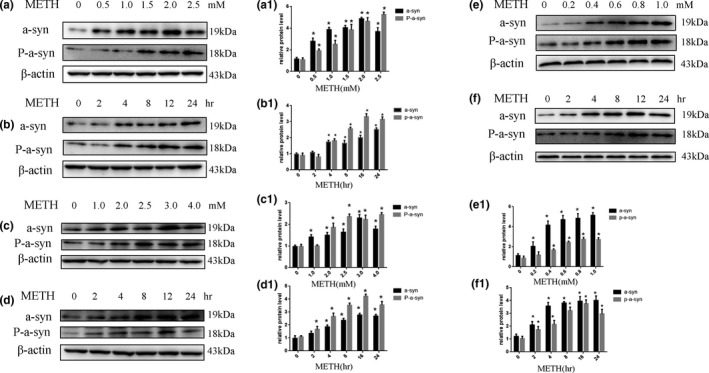
METH exposure upregulates α‐Syn and phosphorylated α‐Syn expression in a concentration‐ and time‐dependent manner in SH‐SY5Y cells, PC12 cells, and primary neuronal cells. SH‐SY5Y cells were exposed to 0–2.5 mM METH for 24 hr (a and a1) and 2.0 mM METH for 2–24 hr (b and b1). PC12 cells were exposed to 0–4.0 mM METH for 24 hr (c and c1) and 3.0 mM METH for 2–24 hr (d and d1). Primary neuronal cells were exposed to 0–1.0 mM METH for 24 hr (e and e1) and 1.0 mM METH for 2–24 hr (f and f1). Western blot and quantitative analyses were performed to determine α‐Syn and phosphorylated α‐Syn protein expression. β‐actin was used as a loading control. *p < 0.05 compared with the control group. The data were analyzed by one‐way ANOVA followed by least significant difference (LSD) *post hoc* analyses. The data are expressed as the means ± standard deviation (*SD*; *n* = 3/group)

Our previous study showed that α‐Syn expression is significantly increased after METH exposure in SH‐SY5Y cells (Chen et al., 2013). To further evaluate how METH affects the expression of α‐Syn and its pathological forms, SH‐SY5Y cells were treated with a dose range (0, 0.5, 1.0, 1.5, 2.0, and 2.5 mM) of METH for 24 hr or treated with 2.0 mM METH for 2, 4, 8, 16, and 24 hr. Western blot analysis was then performed to detect the expression of α‐Syn and its pathological forms. Our results revealed that the expression of α‐Syn and phosphorylated α‐Syn was significantly increased in the METH‐treated SH‐SY5Y cells compared to the controls both in a dose‐ and time‐dependent manner. For instance, after 24‐hr exposure, α‐Syn expression was significantly increased by ~ 2.4‐fold in the METH‐treated (2.0 mM) SH‐SY5Y cells (*n* = 3, *p* < 0.05) (Figure 1a,a1). Similarly, phosphorylated α‐Syn expression was significantly increased by ~3.2‐fold in the cells treated with METH (2.0 mM) for 24 hr (*n* = 3, *p* < 0.05) (Figure 1b,b1). Similar effects were observed in PC12 cells (Figure 1c,d,c1,d1) and primary neurons (Figure 1e,f,e1,f1). Collectively, these data demonstrate that METH exposure induces α‐Syn expression and its abnormal aggregation in neuronal cells.

### 3.2 CMA activity is decreased after METH exposure in neurons

**Figure 2:**
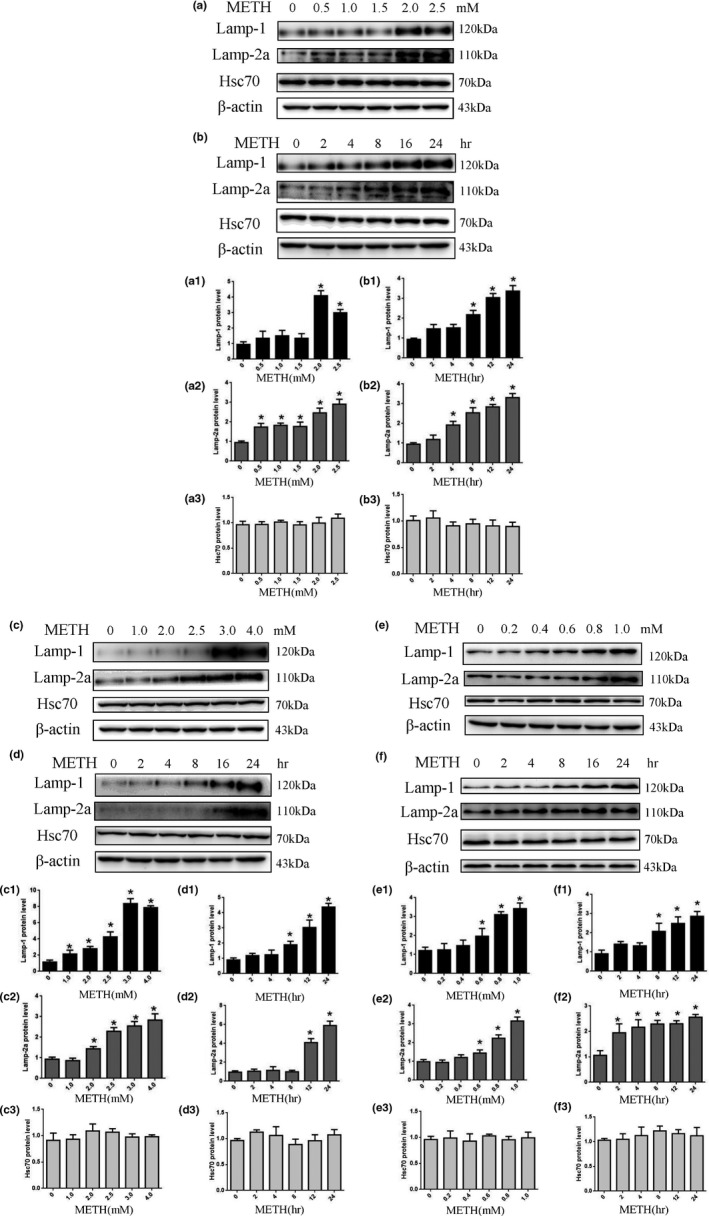
METH exposure upregulates Lamp‐1 and Lamp‐2a expression in a concentration‐ or time‐dependent manner but does not affect the expression of Hsc70 in SH‐SY5Y cells, PC12 cells, and primary neuronal cells. SH‐SY5Y cells were exposed to 0–2.5 mM METH for 24 hr (a and a1–a3) and 2.0 mM METH for 2–24 hr (b and b1–b3). PC12 cells were exposed to 0–4.0 mM METH for 24 hr (c and c1–c3) and 3.0 mM METH for 2–24 hr (d and d1–3). Primary neuronal cells were exposed to 0–1.0 mM METH for 24 hr (e and e1–3) and 1.0 mM METH for 2–24 hr (f and f1–3). Western blot and quantitative analyses were performed to determine Lamp‐1, Lamp‐2a, and Hsc70 protein expression. β‐actin was used as a loading control. *p < 0.05 compared with the control group. The data were analyzed by one‐way ANOVA followed by least significant difference (LSD) *post hoc* analyses. The data are expressed as the means ± standard deviation (*SD*; *n* = 3/group)

**Figure 3:**
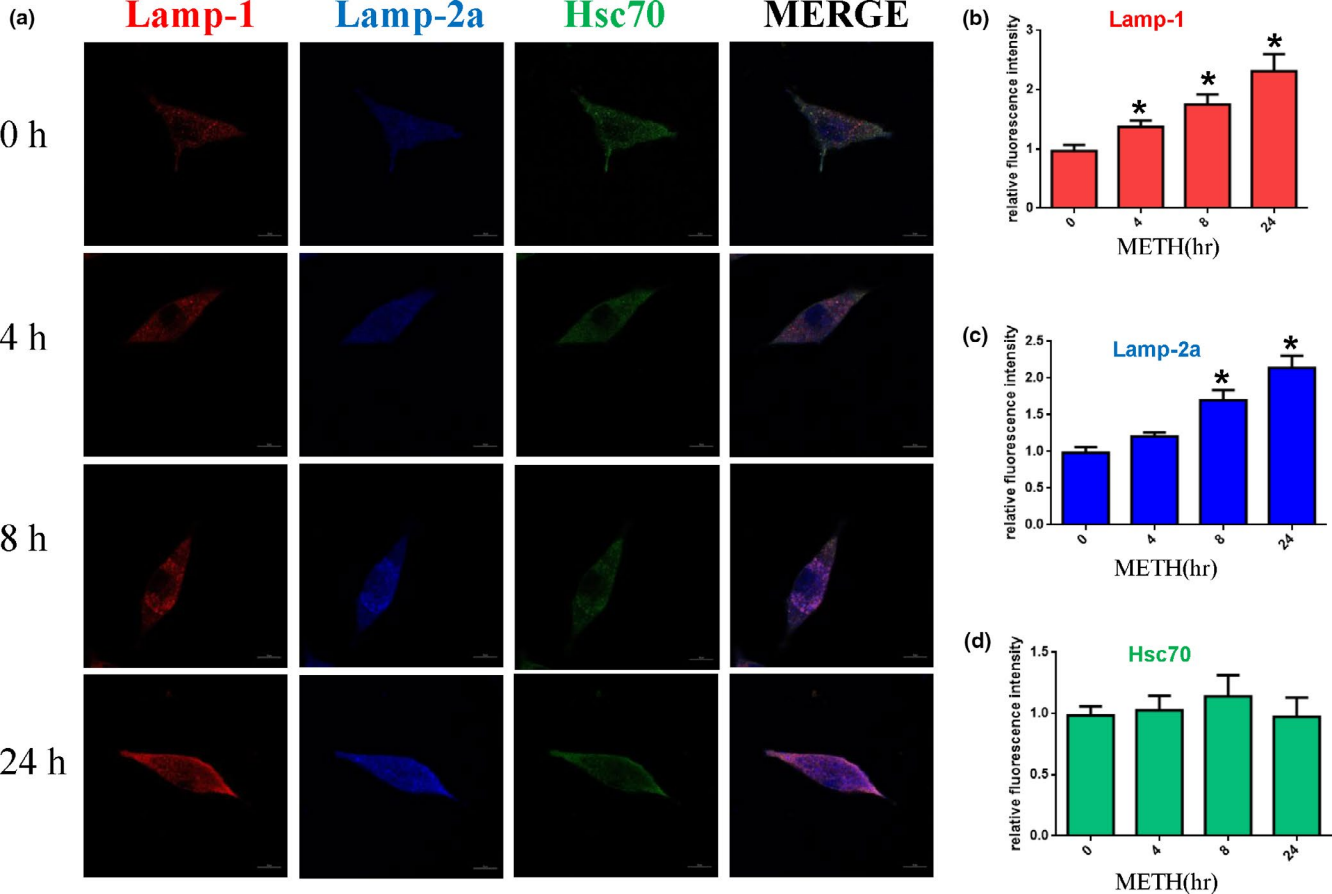
CMA activity was decreased after METH exposure. SH‐SY5Y cells were exposed to 2.0 mM METH for 0, 4, 8, and 24 hr, and CMA activity was assessed by triple immunofluorescence labeling of Lamp‐1 (red), Lamp‐2a (blue), and Hsc70 (green) (a). The expression levels of Hsc70 and Lamp‐2a and Lamp‐1 were determined by the standard quantitative analysis of fluorescence intensity (b1–3). The fluorescence intensity level is presented as the mean ± *SD* (*n* = 3/group). *p < 0.05 compared with the control group

To determine the role of CMA in METH‐triggered neurotoxicity, we assessed the expression levels of CMA‐related protein markers (Lamp‐2a and Hsc70) and a general lysosomal marker (Lamp‐1) after METH exposure. The results showed that 24 hr of METH exposure increased Lamp‐1 and Lamp‐2a expression in a dose‐dependent (Figure 2a,a1,a2) and time‐dependent (Figure 2b,b1,b2) manner. For example, Lamp‐1 and Lamp‐2a expression was ~3.5‐fold and ~3.4‐fold higher, respectively, in the SH‐SY5Y cells treated with 2.0 mM METH for 24 hr compared to that in the control. Additionally, after 4‐hr exposure to 2.0 mM METH, Lamp‐1 and Lamp‐2a protein expression was significantly increased (*n* = 3, *p* < 0.05), and this effect was the largest at 24 hr. Interestingly, there were no substantial changes in the expression level of Hsc70 in METH treatment in either a dose‐ or time‐dependent manner (Figure 2a,a3,b,b3). Similar effects were observed in PC12 cells (Figure 2c,d,c1–3,d1–3) and primary neurons (Figure 2e,f,e1–3,f1–3). Furthermore, we analyzed the coexpression level of Hsc70 with lysosomal markers (Lamp‐1, Lamp‐2a) by immunofluorescence to evaluate CMA activity because both Lamp‐2a and lys‐Hsc70 are limiting factors of CMA (Agarraberes et al., 1997; Cuervo et al., 1997). With the increased expression of Lamp‐1 (Figure 3b) and Lamp‐2a (Figure 3c) and stable Hsc70 (Figure 3d) expression, the expression of Hsc70, compared with Lamp‐1 and Lamp‐2a, was decreased after treatment with 2.0 mM METH for 0, 4, 8, and 24 hr (Figure 3a), indicating that there were not sufficient chaperones involved in the degradation of α‐Syn and that CMA activity was decreased after METH exposure.

### 3.3 Silencing Lamp‐2a expression increases METH‐induced neurotoxicity

**Figure 4:**
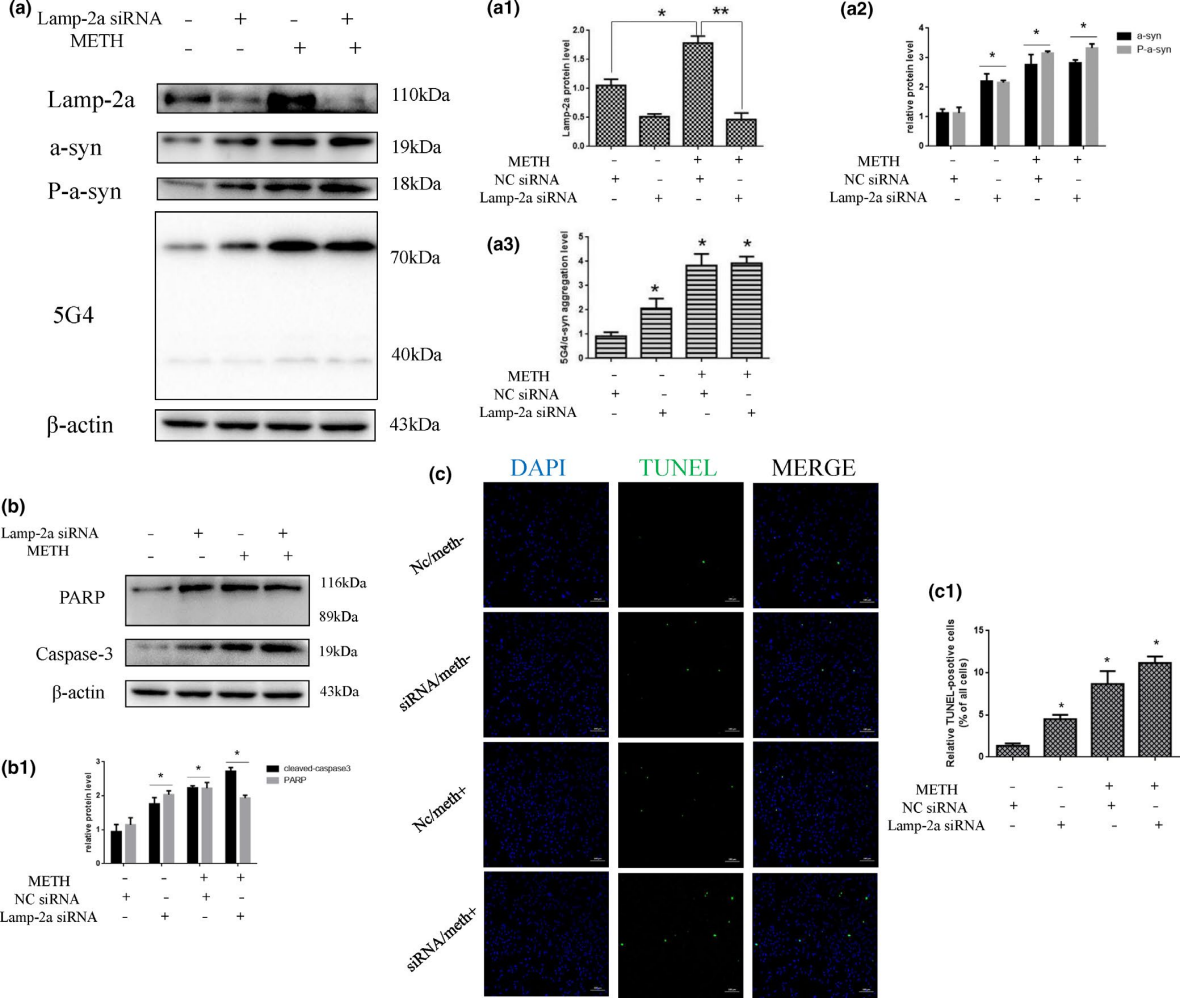
Silencing of Lamp‐2a expression increases METH‐induced neurotoxicity in SH‐SY5Y cells. SH‐SY5Y cells were transfected with NC siRNA or Lamp‐2a siRNA and then treated with or without METH (2.0 mM) for 24 hr. Western blot and quantitative analyses were performed to evaluate the efficiency of Lamp‐2a knockdown and the expression of α‐Syn, phosphorylated α‐Syn, 5G4 (a, a1–3), and the apoptosis markers PARP and cleaved caspase‐3 (b, b1) in SH‐SY5Y cells. β‐actin was used as a loading control. Apoptotic cells were stained with TUNEL (green). Nuclei were counterstained with DAPI (blue) (c). The quantitative analysis of the percentage of apoptotic cells was performed by a standard cell counting method (c1). The number of positive cells is presented as the mean ± *SD* (*n* = 3/group). *p < 0.05 compared with the control group. **p < 0.05 compared with the Lamp‐2a siRNA‐ and METH‐treated group. The data were analyzed by one‐way ANOVA followed by LSD *post hoc* analyses. The data are expressed as the means ± standard deviation (*SD*; *n* = 3/group)

**Figure 5:**
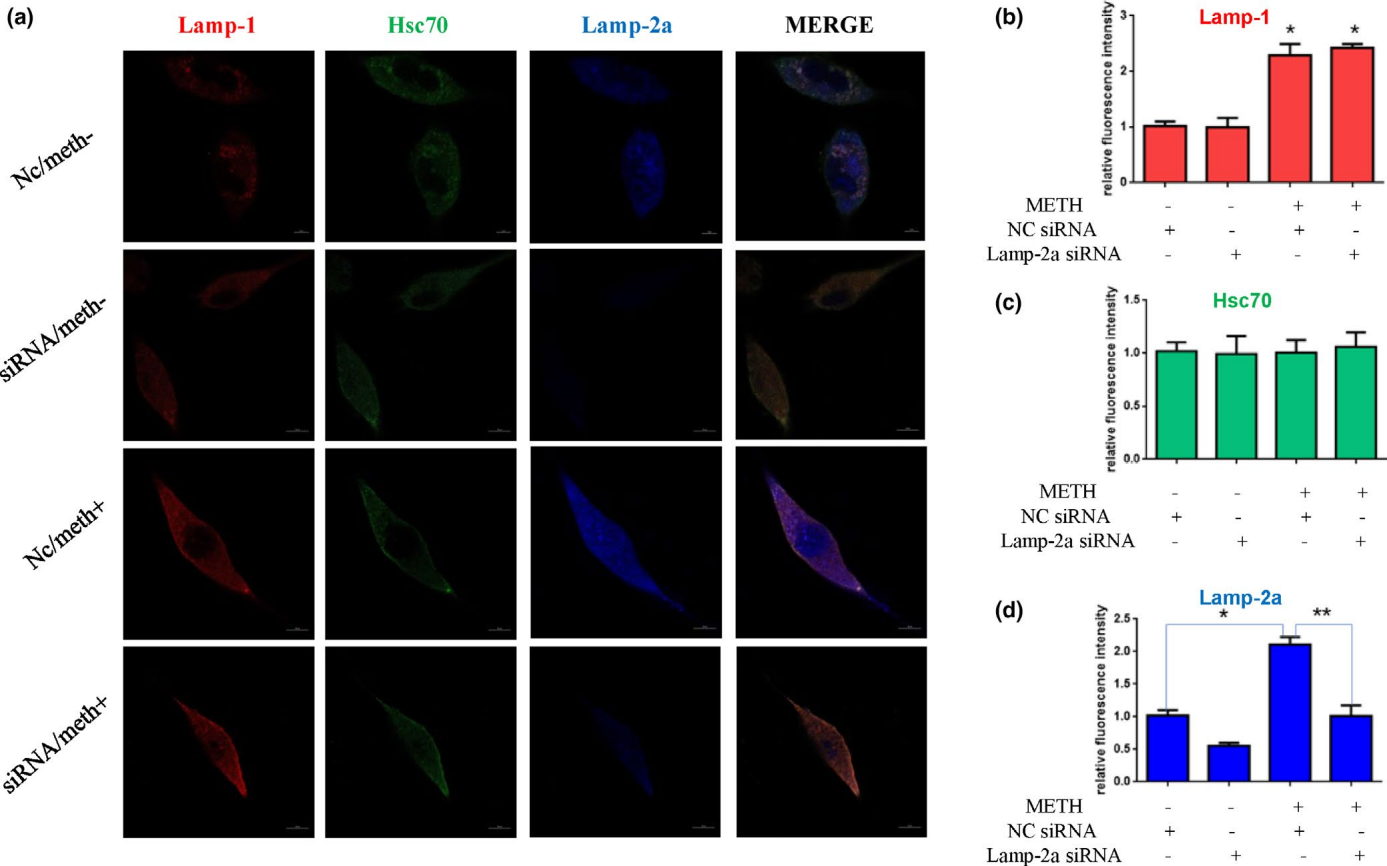
CMA activity was assessed by triple immunofluorescence labeling of Lamp‐1 (red), Lamp‐2a (blue), and Hsc70 (green) (a), and the expression level of Hsc70 and Lamp‐2a and Lamp‐1 was determined by the standard quantitative analysis of fluorescence intensity (b–d). The fluorescence intensity level is presented as the mean ± *SD* (*n* = 3/group). *p < 0.05 compared with the control group. **p < 0.05 compared with the Lamp‐2a siRNA‐ and METH‐treated group

To determine whether Lamp‐2a is involved in METH‐induced neurotoxicity, we designed siRNA sequences to silence Lamp‐2a expression in SH‐SY5Y cells and investigated the impacts on the expression of α‐Syn and its abnormal aggregate forms, CMA activity, and apoptosis triggered by METH using Western blot analysis, immunofluorescence, and TUNEL staining. Western blot analysis showed that Lamp‐2a protein expression was decreased significantly by ~ 76% in the cells exposed to 2.0 mM METH combined with siRNA for 24 hr compared to the cells exposed to METH only (Figure 4a,a1). Additionally, we observed that siRNA exposure significantly increased the expression levels of several proteins, including α‐Syn, phosphorylated α‐Syn (Figure 4a2), 5G4 (Figure 4a3), and the cell apoptosis markers PARP and cleaved caspase‐3 (Figure 4b,b1), compared with that induced by control treatment. TUNEL staining showed that the number of TUNEL‐positive cells was increased by more than ~ 6‐fold in the SH‐SY5Y cells transfected with siRNA compared with the control cells, and the number of TUNEL‐positive cells was also increased both in the cells coexposed to METH and siRNA and in the cells exposed to METH only (Figure 4c,c1). In addition, the expression levels of Lamp‐1 (Figure 5b), Hsc70 (Figure 5c), and Lamp‐2a (Figure 5d), as determined by immunofluorescence, indicated that CMA activity was decreased after siRNA exposure because both Lamp‐2a and lys‐Hsc70 were lacking (Figure 5a). Taken together, these results suggest that silencing Lamp‐2a can significantly reduce CMA activity and exacerbate the neurotoxicity caused by METH exposure in vitro.

### 3.4 Upregulating Hsc70 expression attenuates METH‐induced neurotoxicity

**Figure 6:**
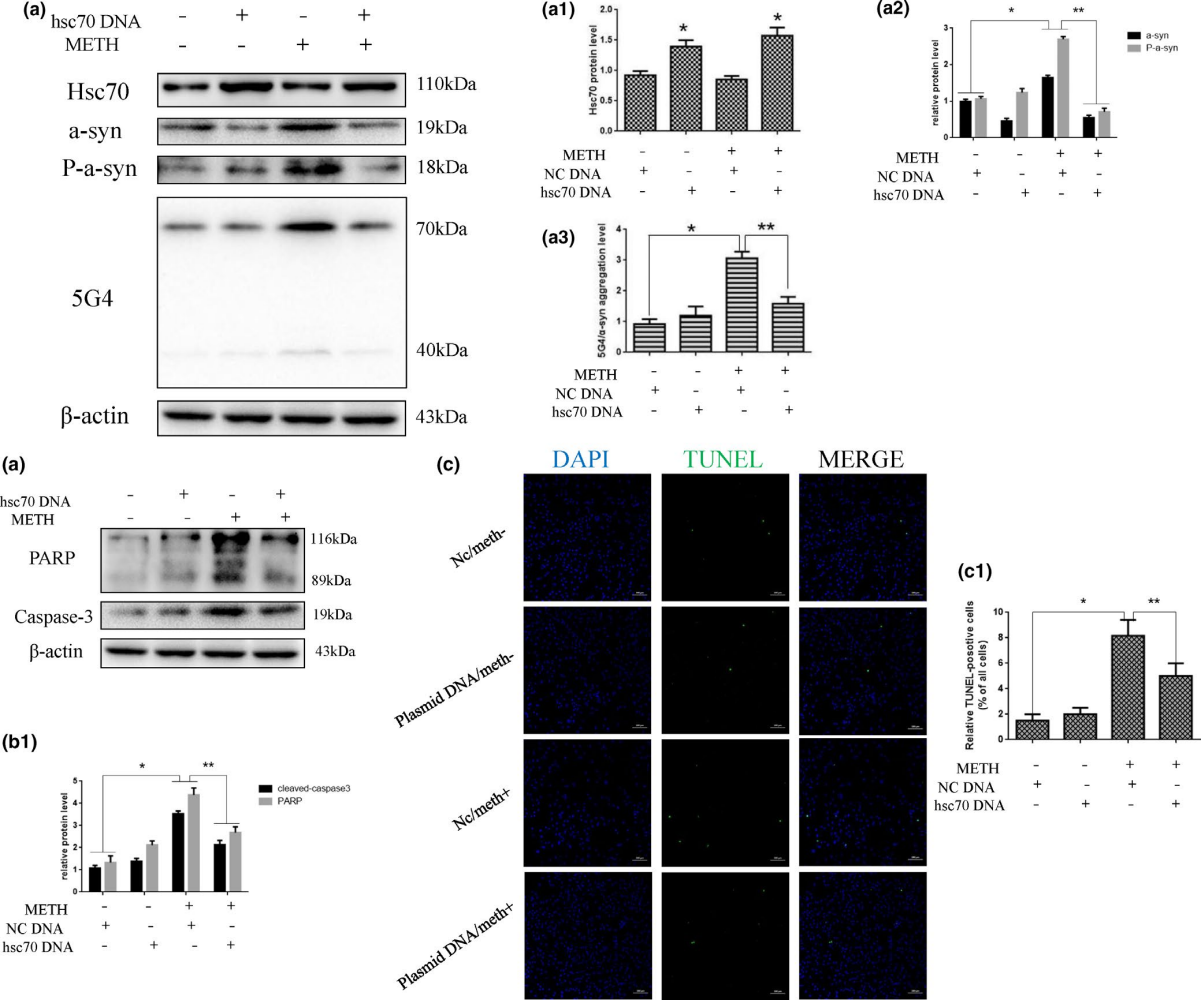
Upregulating Hsc70 expression attenuates METH‐induced neurotoxicity in SH‐SY5Y cells. SH‐SY5Y cells were transfected with an NC plasmid or an Hsc70 overexpression plasmid and then treated with or without METH (2.0 mM) for 24 hr. Western blot and quantitative analyses were performed to evaluate the efficiency of Lamp‐2a knockdown and the expression of α‐Syn, phosphorylated α‐Syn, 5G4 (a, a1–3), and the apoptosis markers PARP and cleaved caspase‐3 (b, b1) in SH‐SY5Y cells. β‐actin was used as a loading control. Apoptotic cells were stained with TUNEL (green). Nuclei were counterstained with DAPI (blue) (c). The quantitative analysis of the percentage of apoptotic cells was performed by a standard cell counting method (c1). The number of positive cells is presented as the mean ± *SD* (*n* = 3/group). *p < 0.05 compared with the control group. **p < 0.05 compared with the Hsc70 overexpression plasmid‐ and METH‐treated group. The data were analyzed by one‐way ANOVA followed by LSD post hoc analyses. The data are expressed as the means ± standard deviation (*SD*; *n* = 3/group)

**Figure 7:**
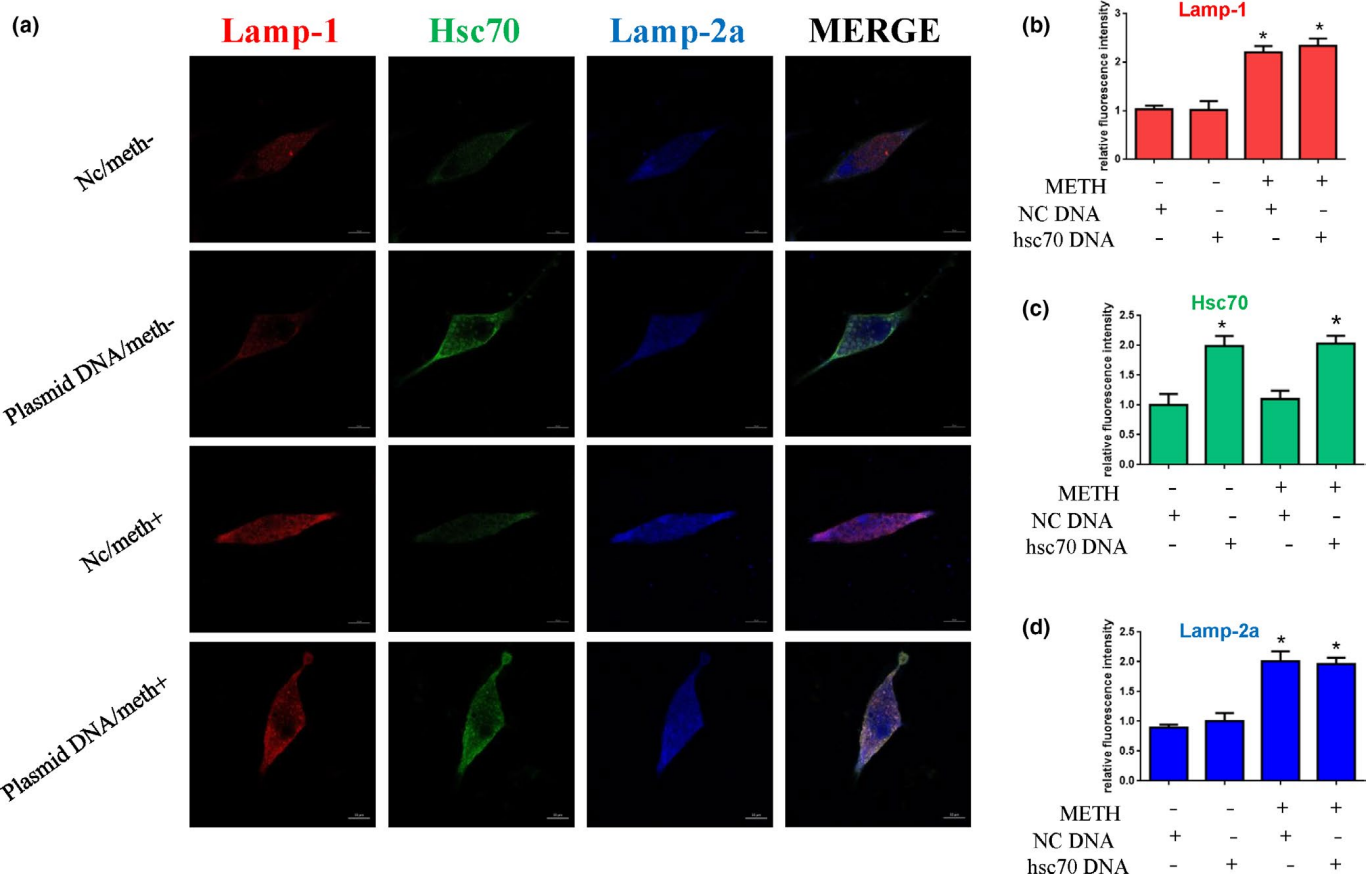
CMA activity was assessed by triple immunofluorescence labeling of Lamp‐1 (red), Lamp‐2a (blue), and Hsc70 (green) (a), and the expression levels of Hsc70 and Lamp‐2a and Lamp‐1 were determined by the standard quantitative analysis of fluorescence intensity (b–d). The fluorescence intensity level is presented as the mean ± *SD* (*n* = 3/group). *p < 0.05 compared with the control group. **p < 0.05 compared with the Hsc70 overexpression plasmid‐ and METH‐treated group

To further assess the role of Hsc70 in METH‐induced neurotoxicity, we investigated whether Hsc70 overexpression affects the aspects of METH‐induced neurotoxicity described above. Western blot analyses showed that Hsc70 expression was increased ~ 1.5‐fold in the plasmid‐treated cells compared with the control cells (Figure 6a,a1). Additionally, the expression of α‐Syn, phosphorylated α‐Syn (Figure 6a2), 5G4 (Figure 6a3), and the cell apoptosis markers PARP and cleaved caspase‐3 (Figure 6b,b1) was significantly increased in the METH‐treated SH‐SY5Y cells compared with the vehicle‐treated cells, and this effect was attenuated by coexposure to the Hsc70 overexpression plasmid. For example, the expression levels of α‐Syn, phosphorylated α‐Syn, and 5G4 were decreased by ~ 75%, ~ 74%, and ~ 45%, respectively, and the expression levels of the cell apoptosis markers were decreased by ~ 35% each. The number of TUNEL‐positive cells was increased by more than 6‐fold in the METH‐treated group compared with the control group, and Hsc70 overexpression plasmid treatment decreased the number of TUNEL‐positive METH‐exposed cells by ~ 50% (Figure 6c,c1). Furthermore, immunofluorescence analyses showed that the expression levels of Lamp‐1 (Figure 7b), Hsc70 (Figure 7c), and Lamp‐2a (Figure 7d) were increased in the cells coexposed to METH and the Hsc70 overexpression plasmid compared with the cells only exposed to METH (Figure 7a).

## 4 DISCUSSION

In this study, we report that the expression of Lamp‐2a is increased after METH exposure, while Hsc70 expression levels do not significantly change in SH‐SY5Y, PC12, and primary neuronal cells. We also demonstrate that the knockdown of Lamp‐2a expression using synthetic siRNA sequences can increase METH‐induced neurotoxicity by increasing the expression of α‐Syn and its abnormal aggregate forms. At the same time, we found that upregulating Hsc70 expression by synthetic plasmid DNA can attenuate the neurotoxicity induced by METH by relieving α‐Syn aggregation. These findings, together with our previous study (Chen et al., 2013), indicate that CMA plays a vital role in METH‐induced toxicity and that the manipulation of CMA may represent a valuable potential therapeutic approach for METH‐induced/‐like neurotoxicity.

In contrast to the other two lysosomal pathways, namely, macroautophagy and microautophagy, CMA can selectively degrade proteins that directly reach the lysosomal lumen without vesicle formation (Cuervo, 2010). Previous studies have identified CMA as a process important for the damage and disease of the central nervous system (Nikoletopoulou, Papandreou, & Tavernarakis, 2015) and have determined that it participates in the pathological mechanisms of neurodegenerative diseases (Cuervo et al., 2004; Malkus & Ischiropoulos, 2012). Studies (Callaghan et al., 2012; Kousik, Carvey, & Napier, 2014) have already shown that METH treatment is extensively used as a model of drug‐induced Parkinsonism because it can cause a PD‐like neuropathology. Additionally, the expression of α‐Syn, which is closely correlated with PD, is significantly increased in SH‐SY5Y cells after METH exposure (Chen et al., 2013). CMA is the main degradation pathway of α‐Syn (Mak et al., 2010; Vogiatzi et al., 2008) and only degrades α‐Syn monomers and dimers, but not oligomers (Martinez‐Vicente et al., 2008). Phosphorylated, dopamine‐modified, and A53T and A30P mutant forms of α‐Syn cannot be degraded through CMA either, and these pathogenic α‐synuclein mutants can be recognized by cytosolic Hsc70 and delivered to the lysosomal membrane; instead, they bind to Lamp‐2a with abnormally high affinity and prevent not only their own degradation but also the CMA‐dependent degradation of other substrates (Cuervo et al., 2004; Martinez‐Vicente et al., 2008). Additionally, the persistence of lysosomal membrane‐bound pathogenic α‐syn promotes its aggregation into toxic oligomers that further impair CMA activity, so CMA can be directly targeted by the toxic effects of α‐syn and generate a bidirectional vicious cycle leading to neuronal demise.

CMA activity can be modulated by the expression of Lamp‐2a, which is the rate‐limiting step (Cuervo & Dice, 2000), and the up‐ or downregulation of Lamp‐2a can significantly influence the expression, posttranslational modification, and high‐molecular‐weight forms of α‐Syn (Vogiatzi et al., 2008; Xilouri et al., 2013). As two other members of the A family, Lamp‐2b and Lamp‐2c can compensate for the other functions of Lamp‐2a in lysosomes, so the genetic reduction of Lamp‐2a levels does not affect lysosomal stability and function but only decreases CMA (Massey, Kaushik, Sovak, Kiffin, & Cuervo, 2006; Schneider, Suh, & Cuervo, 2014; Valdor et al., 2014). In this study, we found that the induction of Lamp‐2a expression by METH occurred in a dose‐ and time‐dependent manner. Thus, we downregulated Lamp‐2a using synthetic siRNA sequences and found that METH‐induced neurotoxicity and the expression of α‐Syn and its abnormal aggregate forms were increased. We also found that changes in Hsc70 expression were not significantly affected in a dose‐ or time‐dependent manner in response to METH treatment. Human Hsc70 and Hsp70 share 85% primary structural identity, and it has been thought that they play similar cellular roles (Goldfarb et al., 2006; Hageman, van Waarde, Zylicz, Walerych, & Kampinga, 2011). Both of them have been shown to prevent α‐Syn fibril formation, protect against α‐Syn toxicity, and reduce the number of α‐Syn aggregates (Dedmon, Christodoulou, Wilson, & Dobson, 2005; Gao et al., 2015; Klucken, Shin, Masliah, Hyman, & McLean, 2004; Opazo, Krenz, Heermann, Schulz, & Falkenburger, 2008; Pemberton et al., 2011; Pemberton & Melki, 2012). However, Hsp70 overexpression has been shown to neither influence the formation of α‐Syn oligomeric species nor counteract the nonbeneficial motor deficits associated with α‐synucleinopathies (Shimshek, Mueller, Wiessner, Schweizer, van der Putten, 2010). However, Hsc70 and its cochaperones from the Hsp40 family confront high‐molecular‐weight α‐Syn assemblies prior to Hsp70. Thus, Hsc70 is critical in the early stages of α‐Syn aggregation (Pemberton et al., 2011). Additionally, because Hsc70 has other important cellular functions, the blockage of Hsc70 affects not only CMA but also endosomal microautophagy (e‐MI), macroautophagy, and endocytosis, as well as the processes of protein folding and aggregation (Stricher et al., 2013). Thus, we upregulated Hsc70 by synthetic plasmid DNA. Interestingly, the neurotoxicity induced by METH and the aggregation of α‐Syn were relieved after Hsc70 overexpression. Therefore, we conclude that CMA plays a vital role in METH‐induced neurotoxicity.

The activity of CMA is tightly linked with changes in key CMA components, such as Lamp‐2a and Hsc70, and in the amount and distribution of CMA‐active lysosomes. Previous studies have shown that both Lamp‐2a and lys‐Hsc70 are limiting factors in the modulation of CMA (Agarraberes et al., 1997; Cuervo et al., 1997). Thus, to elucidate the change in CMA activity, we analyzed the expression level of Hsc70 and lysosomal markers (Lamp‐1, Lamp‐2a) by immunofluorescence as described previously (Cuervo et al., 1997; Kaushik & Cuervo, 2009; Kiffin, Christian, Knecht, & Cuervo, 2004). We found that CMA activity is decreased by a relatively high dose of METH due to a lack of Hsc70 and is aggravated by Lamp‐2a silencing, whereas it is recovered by Hsc70 overexpression.

In conclusion, our present study highlights the fact that CMA plays a pivotal role in METH‐induced neurotoxicity. We demonstrate that the Hsc70 level is not significantly changed after METH treatment in SH‐SY5Y, PC12, and primary neuronal cells, and the upregulation of Hsc70 expression significantly protects neuronal cells against METH‐induced toxicity, mainly reflected by the restoration of CMA activity and the decreased expression of α‐Syn and its abnormal aggregate forms, followed by reduced apoptosis. These findings indicate the therapeutic potential of targeting this pathway for the treatment of METH abuse and neurodegenerative disorders. Further studies are needed to determine the exact protective mechanisms of CMA in neurotoxicity induced by METH in vivo.

## Data Availability

The data that support the findings of this study are openly available in figshare at http://doi.org/10.6084/m9.figshare.8166029 (Sun, 2019).
